# Clinical Implications of Tumor-Infiltrating Immune Cells in Breast Cancer

**DOI:** 10.7150/jca.35901

**Published:** 2019-10-15

**Authors:** Shi-Chao Zhang, Zu-Quan Hu, Jin-Hua Long, Gui-Ming Zhu, Yun Wang, Yi Jia, Jing Zhou, Yan Ouyang, Zhu Zeng

**Affiliations:** 1 Immune Cells and Antibody Engineering Research Center of Guizhou Province, Key Laboratory of Biology and Medical Engineering, Guizhou Medical University, Guiyang 550025, China; 2 Engineering Research Center of Medical Biotechnology, School of Biology and Engineering, Guizhou Medical University, Guiyang 550025, China; 3 School of Basic Medical Science, Guizhou Medical University, Guiyang 550025, China; 4 Affiliated Tumor Hospital, Guizhou Medical University, Guiyang 550004, China

**Keywords:** breast cancer, tumor-infiltrating immune cells (TIICs), prognosis, disease-free survival (DFS), overall survival (OS)

## Abstract

The immune infiltration of tumors is closely related to clinical outcomes. The composition of tumor-infiltrating immune cells (TIICs) can serve as biomarkers for predicting response to treatment and survival in different patient subgroups in terms of chemotherapy and immunotherapy. This study is focused on investigating the clinical implications of TIICs in breast cancer patients. We performed several *in silico* analyses of gene expression profiles in 2976 nonmetastatic tumor samples. CIBERSORT was used to estimate the proportion of 22 immune cell types to analyze their correlation with overall survival (OS) and disease-free survival (DFS) in different breast cancer subtypes and stages. Our results showed that a higher fraction of plasma cells in estrogen receptor (ER)-positive breast cancer patients indicated an increase in DFS (hazard ratio [HR]=0.66, 95% confidence interval [CI] 0.54~0.82, *p*<0.01), while a decreased OS was correlated with a greater number of M0 macrophages (HR=2.02, 95% CI 1.27~3.30, *p*=0.01) and regulatory T cells (HR=1.90, 95% CI 1.20~3.02,* p*=0.02). In ER-negative or progesterone receptor (PR)-negative subtypes or in a combined subtype, the increase in activated memory CD4^+^ T cells was correlated with increased DFS (HR=0.46, 95% CI 0.33~0.63,* p*<0.01). In all breast cancer patients, a higher proportion of M0 macrophages indicated a decreased DFS (HR=1.67, 95% CI 1.22~2.27, *p*<0.01), while increased OS was associated with relatively larger fractions of resting memory CD4^+^ T cells (HR=0.70, 95% CI 0.55~0.90, *p*=0.02) and γδ T cells (HR=0.66, 95% CI 0.51~0.85, *p*<0.01). Therefore, this study revealed that the composition of TIICs is different in patients with various subtypes of breast cancer and is directly related to prognosis, suggesting that TIICs are important participants in tumor progression and may, potentially be used for future diagnosis and treatment.

## Background

Breast cancer is the most frequent and common malignant tumor and the leading cause of cancer death among females 1. Moreover, it is a heterogeneous disease with high clinical diversity, and thus, the standard treatment regimens are not suitable for all patients 2. In recent years, great advances have been achieved in early diagnosis, surgical techniques, neoadjuvant and adjuvant treatments, but the clinical outcomes are still unsatisfactory 3. Therefore, it is essential to predict treatment responses, select more personalized treatment regimens, and explore new drug targets for different patient subgroups 4.

Tumor-infiltrating immune cells (TIICs) are indispensable components of the tumor microenvironment and have been used for the prediction of prognosis and treatment in cancer patients 4-6. Tumor-infiltrating lymphocytes (TILs) are the most widely studied populations of TIICs and their correlation with favorable outcomes in breast cancer has been reported previously 7-9. In patients with human epidermal growth factor receptor 2 (HER2)-positive tumors, a high number of TILs is associated with increased overall survival (OS) and may predict an improved response to anthracyclines and trastuzumab 9-11. Moreover, the presence of TILs can decrease distal recurrence and increase metastasis-free survival in naive triple-negative breast cancer 11-14. However, the number of TILs is not always proportional to the treatment response, suggesting that the immune phenotypes of TILs are also an important prognostic factor 7. In addition to TILs, tumor-associated macrophages (TAMs) are another immune population that can regulate the interaction between cancer and the immune system. Clinicopathological studies have suggested that TAM accumulation in tumors usually correlates with a poor clinical outcome 15. Furthermore, there have been some inconsistent opinions regarding the effects of TIICs on cancer patients, which may be due to differences in tumor molecular subtypes between tumor serial progression stages 5-7.

Immunotherapies based on immune checkpoints have significantly improved clinical outcomes in a subset of patients with solid tumors, including breast cancer. Recently, cytotoxic T-lymphocyte-associated protein 4 (CTLA4), programmed death-1 (PD-1) and programmed death-ligand 1 (PD-L1) have been shown to be highly promising targets for modulating the interaction of immune cells and tumor cells. However, successful cases have been limited to specific types of cancers, which has been attributed to the insufficient and heterogeneous expression of checkpoint molecules in the tumor microenvironment 16, 17. Moreover, the distribution of TIICs may an important factor in determining whether patients show a good response to anticancer therapies 18, 19. Previous studies concerning the composition of TIICs have relied mainly on flow cytometry and immunohistochemistry, but these methods can only evaluate a few immune cell types at once and are limited by phenotypic markers 20. Interestingly, the algorithmic analysis of a large amount of gene expression data provides a fast and effective method for estimating the composition of TIICs.

In this study, we performed several *in silico* analyses on the gene expression profiles of 2976 unrelated tumor samples from nonmetastatic breast cancer patients with known clinical follow-up. CIBERSORT 21, 22, an established computational approach, was applied to estimate the relative proportions of 22 types of immune cells in these tumor samples. This is conducive to confirming the cellular composition in terms of the immune response in tumor patients and disclosing the associations among molecular subtypes, clinicopathological variables and survival. In addition, immune checkpoint modulators in breast cancer were analyzed in an attempt to reveal their potential prognostic value.

## Materials and methods

### Data acquisition

Data from the public domain were used in this study. Raw microarray expression data from primary tumors from patients with nonmetastasized breast cancer were downloaded from the Gene Expression Omnibus (GEO). GEO accession numbers of these data were GSE16391, GSE19615, GSE20685, GSE20713, GSE21653, GSE22035, GSE22646, GSE26639, GSE28583, GSE28694, GSE28826, GSE29044, GSE31192, GSE31448, GSE32646, GSE36771, GSE40837, GSE41568, GSE43502, GSE43568, GSE47389, GSE48390, GSE48391, GSE58984, GSE71258, GSE76124, GSE87007, GSE87377, GSE88770. Clinicopathological data were also collected, including gender, age, TNM stage, grade, Ki67 status, tumor size, the use of adjuvant chemotherapy, OS and DFS, and estrogen receptor (ER), progesterone receptor (PR) and HER2 status. To avoid the influence of differences among platforms, our analyses were confined to samples that hybridized to the Affymetrix HG-U133 Plus 2.0 platform (accession number GPL570).

### Estimation of tumor-infiltrating immune cells

CIBERSORT, a deconvolution algorithm, was applied to estimate the abundances of specific cell types in complex tissues based on gene expression profiles, which has been proven to be a method resulting in a high degree of consistency during actual assessment in tumor studies 4, 21, 22. Normalized gene expression data were used to estimate the relative proportions of 22 types of TIICs using CIBERSORT. Simultaneously, the LM22 signature matrix with 1000 permutations was used for the performance of the algorithm. The samples for which the CIBERSORT analysis resulted in *p*<0.05 were used for further analysis. These TIICs included seven types of T cells (resting memory CD4^+^ T cells, activated memory CD4^+^ T cells, CD8^+^ T cells, naive CD4^+^ T cells, γδ T cells, follicular helper T cells, and regulatory T cells), memory B cells, naive B cells, activated mast cells, resting mast cells, activated dendritic cells (DCs), resting DCs, macrophages (M0, M1 and M2), eosinophils, monocytes, activated natural killer (NK) cells, resting NK cells, plasma cells and neutrophils. For each sample, the sum of all estimated immune cell-type fractions equalled 1.

### Statistical analysis

Statistical analyses were conducted using R v3.3.2 and Bioconductor (https://www.bioconductor.org/). The aggregation and preparation of the raw data were performed using the “affy” package. Each dataset was processed by a weighted average method to compare the differences in the compositions of TIICs in different subtypes and grades of breast cancer. We used a univariate linear regression model to assess the association between the percentage of immune cells and clinicopathological variables. Cox regression analysis was performed to evaluate the prognostic values of TIICs and immune checkpoint molecules. The hazard ratio (HR) and 95% confidence interval (CI) were determined, and a *p*-value less than 0.05 was considered statistically significant for all statistical analyses.

## Results

### Data set of breast cancer samples

**Figure 1:**
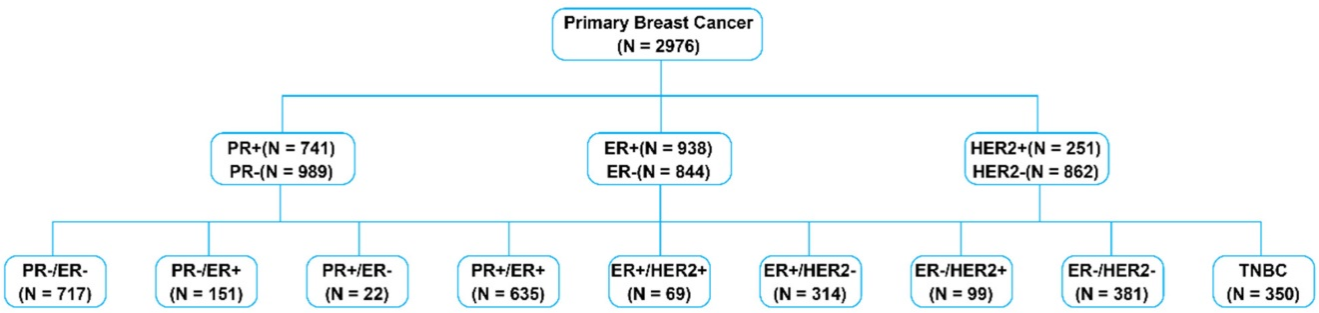
Overview of breast cancer subtypes based on receptor status.

A data set that included 2976 breast cancer samples was chosen for the analyses in this study. A summary of the primary tumor characteristics of these samples is presented in Table 1. Samples were classified according to their ER, PR and HER2 statuses (Figure 1).

### Composition of TIICs

**Figure 2:**
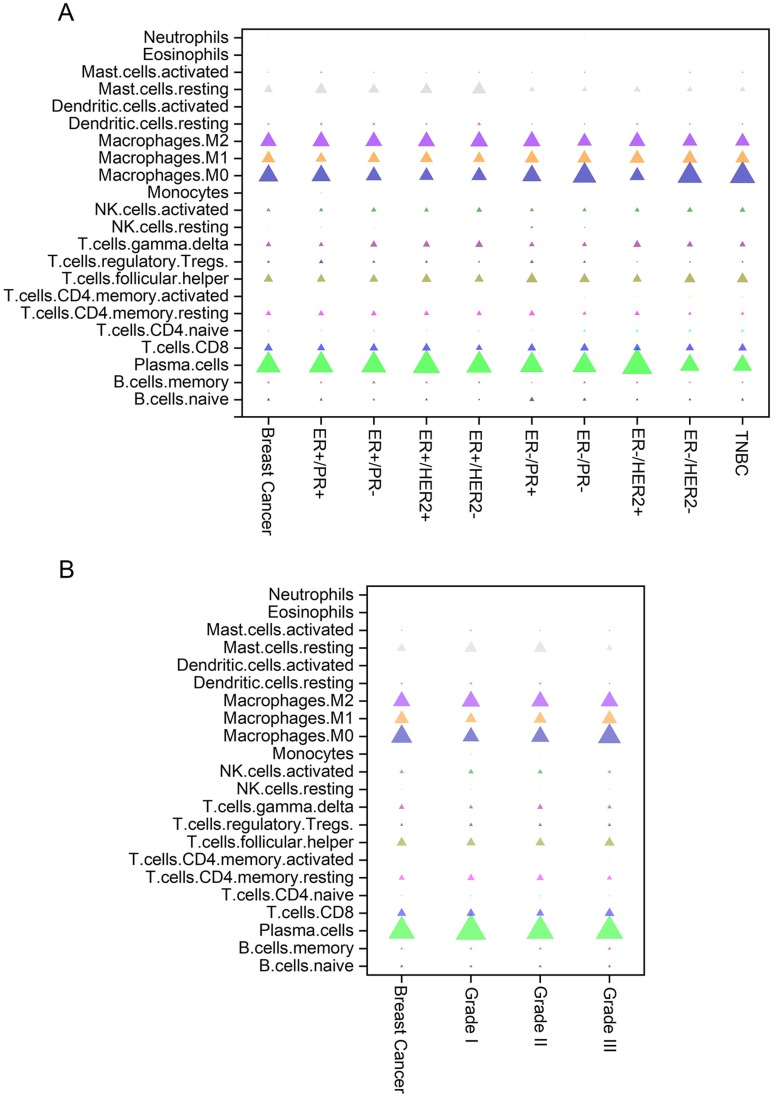
Distribution of immune cell-type frequencies in breast cancer of different subtypes (A) and grades (B). The fractions of each immune cell type in different breast cancer subtypes and stages were compared. The size of the bubble represents the fraction of the immune cell-type.

**Figure 3:**
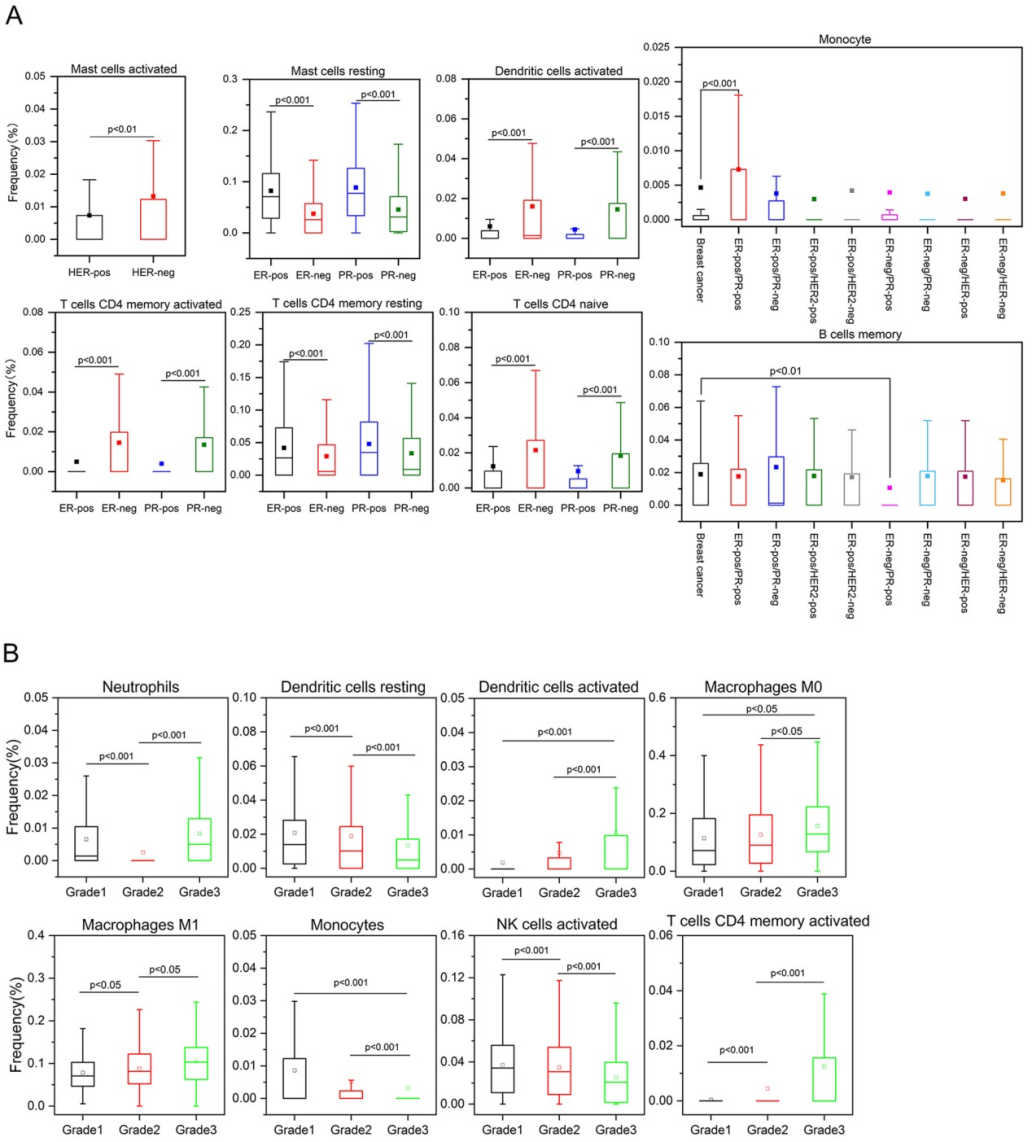
Differences in the composition of TIICs in various subtypes (A) and grades (B) of breast cancer.

The composition of immune cells in breast cancers of different subtypes and grades are shown in Figure 2. Breast tumors generally contained an abundance of TAMs (36.3%), plasma cells (17.9%) and follicular helper T (Tfh) cells (6.9%), whereas eosinophils (0.1%), monocytes (0.5%) and resting NK cells (0.6%) were scarce. The composition of TIICs was greatly different in various subtypes of breast cancer. Compared with HER2-positive tumors, there was a higher proportion of activated mast cells in HER2-negative samples. The number of activated memory CD4^+^ T cells, activated DCs and naive CD4^+^ T cells was increased in ER-negative cancers, while more resting mast cells and resting memory CD4^+^ T cells existed in ER-positive cancers (Figures 2A and 3A). As the tumor grade increased, the proportions of activated DCs, activated memory CD4^+^ T cells, M0 and M1 macrophages were gradually increased, whereas activated NK cells, monocytes, and resting DCs were decreased in cancer patients with higher grade tumors (Figures 2B and 3B).

### Immune cell-type fractions and patient prognosis

**Figure 4:**
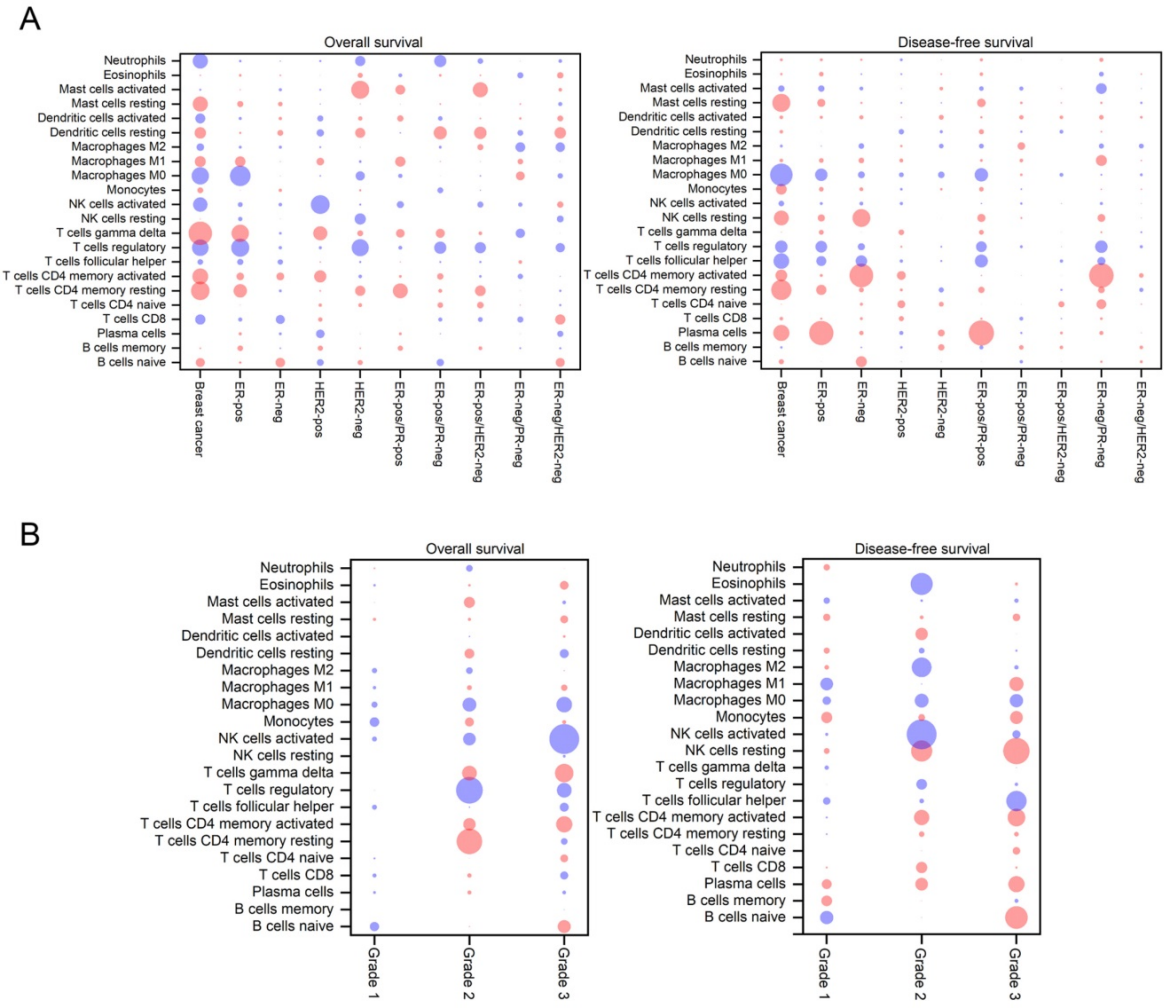
Bubble heat map showing the predictive and prognostic value of immune cell-type frequencies in breast cancer of different subtypes (A) and grades (B). The associations between the fractions and OS and DFS were assessed by Cox regression analysis. A blue bubble indicates that a higher fraction of immune cells is associated with decreased DFS or OS; a red bubble indicates that a higher fraction of immune cells is associated with increased DFS or OS. The size of the bubble indicates the statistical significance level.

To assess the prognostic values of immune cells, a Cox regression analysis was used to analyze the correlations between survival and the proportions of TIICs. As shown in Figure 4, the sizes of the bubbles in the heatmap represent the level of statistical significance, while blue and red colors indicate a negative and positive correlation, respectively, between TIICs and OS/DFS. In breast cancer patients, a higher proportion of M0 macrophages indicated decreased DFS (HR=1.67, 95% CI 1.22~2.27, *p*<0.01), while increased OS was associated with relatively higher fractions of resting memory CD4^+^ T cells (HR=0.70, 95% CI 0.55~0.90, *p*=0.02) and γδ T cells (HR=0.66, 95% CI 0.51~0.85,* p*<0.01). In ER-positive breast cancer patients, an increased fraction of plasma cells indicated an increase in DFS (HR=0.66, 95% CI=0.54~0.82, *p*<0.01), while a decreased OS was correlated with an increased number of M0 macrophages (HR=2.02, 95% CI 1.27~3.30, *p*=0.01) and regulatory T cells (HR=1.90, 95% CI 1.20~3.02,* p*=0.02). Conversely, an increase in activated memory CD4^+^ T cells in ER-negative or PR-negative subtypes or in tumors with a combined subtype was related to an increase in DFS (HR=0.46, 95% CI 0.33~0.63,* p*<0.01). In addition, larger fraction of activated mast cells in HER2-negative tumors was associated with an improved OS (HR=0.30, 95% CI 0.12~0.71,* p*=0.02), while a higher fraction of activated NK cells in HER2- positive patients were associated with decreased OS (HR=6.25, 95% Cl 1.74~22.49, *p*<0.02) (Figure 4A).

In patients with different stages of cancer, the correlation between TIICs and OS/DFS exhibited large differences (Figure 4B). In grade 2 tumors, increased OS was associated with a relatively higher proportion of resting CD4^+^ T cells (HR=0.41, 95% CI 0.21~0.79, *p*=0.03) and a smaller fraction of regulatory T cells (HR=2.40, 95% CI 1.28~4.49, *p*=0.02), while larger fractions of activated NK cells (HR=2.43, 95% CI 1.39~4.23, *p*<0.01), eosinophils (HR=3.75, 95% CI 1.38~10.18, *p*=0.03), and M2 macrophages (HR=1.91, 95% CI 1.13~3.25, *p*=0.04) were correlated with decreased DFS. In grade 3 tumors, increased OS was related to a smaller fraction of activated NK cells (HR=2.16, 95% CI 1.29~3.61, *p*=0.01), while increased DFS was related to higher proportions of resting NK cells (HR=0.60, 95% CI 0.42~0.85, *p*=0.01), naive B cells (HR=0.64, 95% CI 0.45~0.89, *p*=0.03) and a relatively smaller fraction of Tfh cells (HR=1.52, 95% CI 1.08~2.11, *p*=0.04).

### Association between TIICs and clinicopathological variables

**Figure 5:**
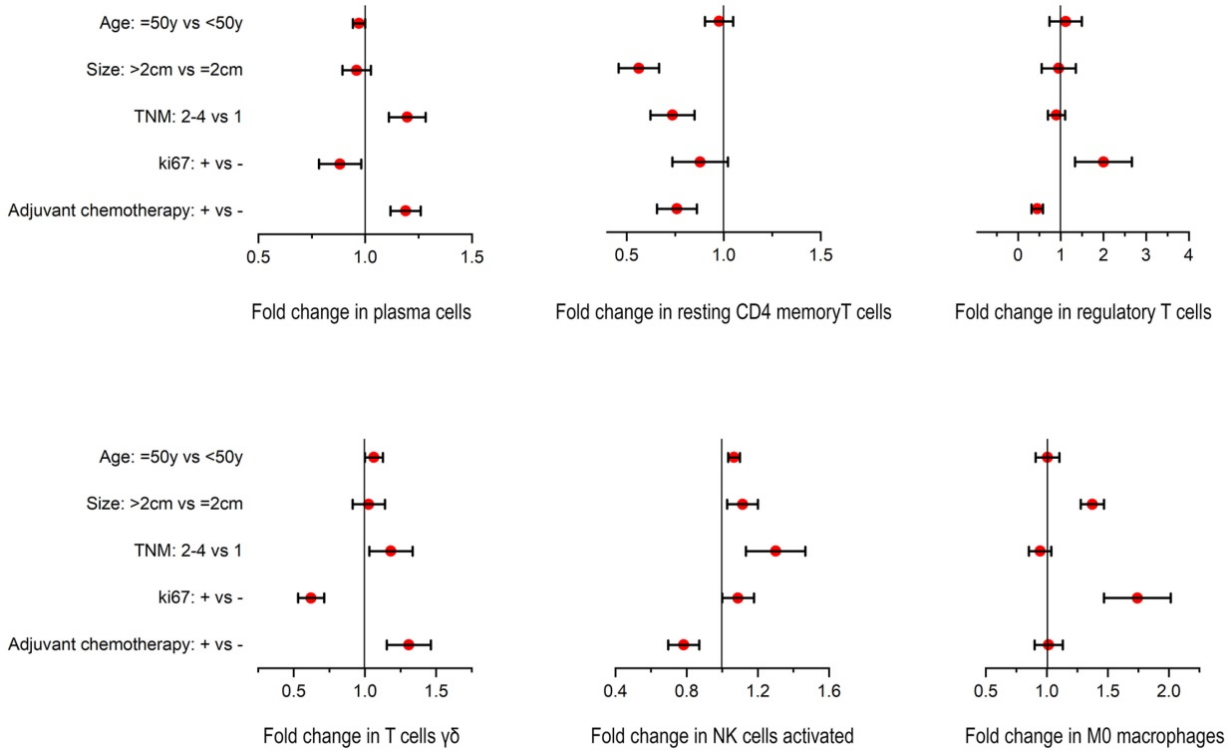
Univariate associations between the composition of TIICs and clinicopathological variables in a breast cancer cohort.

Based on the above analyses, we found that plasma cells, resting memory CD4^+^ T cells, regulatory T cells, γδ T cells, activated NK cells, and M0 macrophages played relatively important roles in breast cancer. Therefore, a univariate analysis was performed to further reveal the potential correlations between these TIICs and clinicopathological variables including age, TNM stage, Ki67 status, tumor size, and the use of adjuvant chemotherapy. As shown in Figure 5, resting memory CD4^+^ T cells were associated with a smaller tumor size. M0 macrophages and regulatory T cells were associated with increased proliferation (≥20% Ki67). In patients receiving adjuvant chemotherapy, high numbers of plasma cells and γδ T cells and a smaller fraction of regulatory T cells were observed.

### Immunomodulators and tumor subtypes

**Figure 6:**
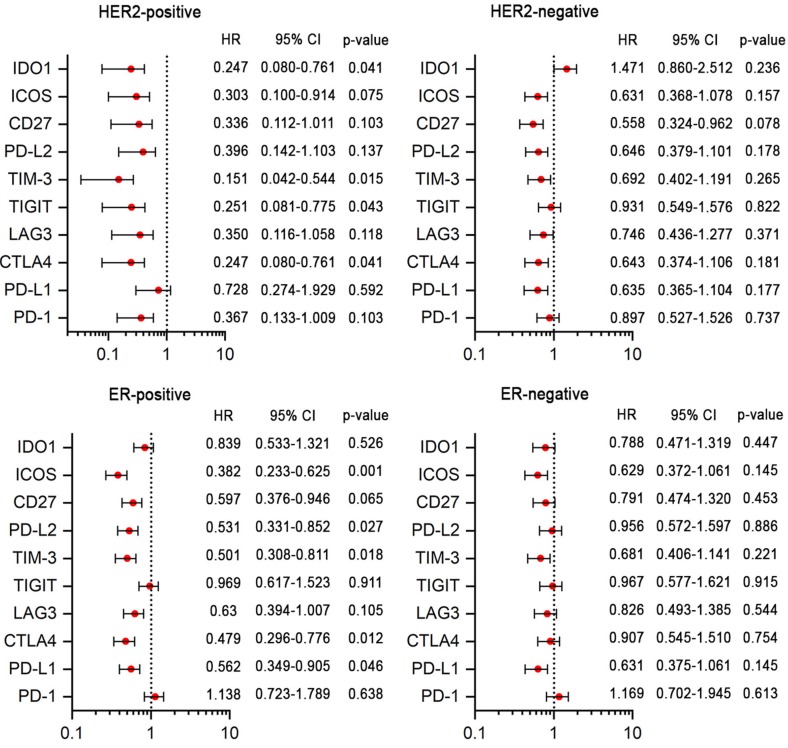
Cox regression analyses of the prognostic value of immunomodulators in ER-positive, ER-negative, HER2-positive and HER2-negative patients.

To evaluate the potential prognostic value of immune checkpoint molecules participating in tumor immune escape, a total of ten immunomodulators were analyzed, including two co-stimulatory molecules (CD27 and ICOS) and eight co-inhibitory molecules (PD-1, TIM-3, LAG3, PD-L1, CTLA4, PD-L2, TIGIT and IDO1) (Figure 6). A poor prognosis was associated with low expression levels of TIM-3, CTLA4, TIGIT and IDO1 in the HER2-positive subtype and with low expression levels of ICOS, TIM-3, PD-L1, CTLA4 and PD-L2 in ER-positive breast cancer patients. Thus, these results indicated that immunotherapies targeting to these immune checkpoint molecules may result in limited or no added value for HER2-negative or ER-negative breast cancer patients.

## Discussion

In this study, we revealed distinct patterns of immune infiltration in various breast cancer patient subgroups based on the deconvolution of bulk gene expression data from a large set of samples. Classically, breast cancer is classified into four subtypes ([ER+|PR+]HER2+, [ER+|PR+]HER2-, ER-PR-HER2+, and ER-PR-HER2-) according to the dichotomized immunohistochemical evaluation of three receptors 23, 24. In our analyses, to obtain much more detailed information concerning the clinical implications of TIICs in breast cancer patients by analyzing ER- and HER2-predominant subtypes, a total of 2976 nonmetastatic breast tumor samples were classified into nine subtypes based on receptor status, including ER+PR+, ER+PR-, ER-PR+, ER-PR-, ER+HER2+, ER+HER2-, ER-HER2+, ER-HER2-, and ER-PR-HER2-. Among them, the proportions of the ER-PR- and ER+PR+ subtypes were greatest, whereas the ER-PR+ subgroup was the smallest, which is consistent with the fact that ER and PR status are strongly associated with each other, since PR-positive status depends on ER-positive status 2, 23. Furthermore, the prognostic significance of TIICs was investigated and the associations between TIICs and clinicopathological parameters were revealed to provide guidance for the development of immune-modulating therapies. We found that the proportion of regulatory T cells was higher in the ER-positive than in the ER-negative subtype, and that an increased fraction of regulatory T cells was related to decreased OS in ER-positive tumors. Regulatory T cells can express inhibitory receptors to maintain self-tolerance and suppress the anti-tumor activity of effector T cells by secreting soluble immunosuppressive cytokines 25, 26. Hence, the anti-CTLA4 antibody (ipilimumab), which can kill effector regulatory T cells or attenuate their suppressive activity, is considered a promising treatment drug 26. Our observations are in line with previous findings that indicate that the regulatory T cells, compared to other immune cell populations, predict statistically significantly worse prognosis for many malignancies 27, 28. In addition, regulatory T cells are decreased after adjuvant chemotherapy, which may profoundly drive the development of tumor-specific CD4^+^ T effector cells. This provides a valuable insight and may aid in the development of a potential intervention strategy for ER-positive subtypes.

Macrophages can be divided into classical M1 and alternative M2 macrophages based on their function 29. It is generally believed that M1 macrophages participate in inflammatory reactions and anti-tumor immunity, while M2 macrophages have pro-tumorigenic properties. However, the “polarized states” of macrophages are controversial. M1 and M2 phenotypes may represent the extremes of a range of functional states rather than truly different cell types 30. In this study, we observed that a high fraction of M0 macrophages were related to decreased OS and DFS in ER-positive tumors. In terms of the development of interventions that can affect macrophage polarization, our results deserve further attention. Moreover, an increased fraction of M0 macrophages was observed in breast tumors with higher grades, which implied that circulating macrophages can be gradually recruited into tumors to alter the tumor microenvironment and promote tumor progression. Thus, our results further suggested that TAMs could be considered biomarkers for breast cancer.

Compared with the ER-positive subtype, ER-negative tumors had a higher fraction of activated memory CD4^+^ T cells, which was related to increased DFS. Memory T cells have a long lifespan, which contributes to their key role in tumor development 31. The subsets of memory T cells are CD4^+^ and CD8^+^ memory T cells. Memory CD8^+^ T cells can kill tumor cells upon secondary recognition of the tumor-associated antigens 32. Meanwhile, memory CD4^+^ T cells suppress the outgrowth of tumor cells by promoting the proliferation of CD8^+^ cells, which migrate to tumor-associated tissues and differentiate into effector cells 33, 34. Our findings showed that increased DFS was directly correlated with an increase in resting and activated memory CD4^+^ T cells in breast cancer, indicating the anti-tumor properties of memory CD4^+^ T cells. Unfortunately, due to the reduction in resting memory CD4^+^ T cells after adjuvant chemotherapy, this clinical strategy may show little efficacy against ER-positive, PR-positive or combined subtypes.

Our results showed that the presence of an increased fraction of activated NK cells suggested a poor prognosis in HER2-positive tumors, especially for breast cancer patients with advanced stage tumors. Occasionally, NK cells exhibit immunoregulatory functions and can limit T cell responses 35, which may contribute to metabolic reprogramming-induced aberrant effector functions 36, 37. Therefore, the interactions of immune cells in the tumor microenvironment are complicated, and chemotherapy-induced reduction of activated NK cells may be favorable for strengthening the overall antitumor immunity in host. B cells can differentiate into antibody-secreting plasma cells and memory B cells upon antigen activation, contributing to humoral immunity 38. Our study found that a higher fraction of plasma cells was associated with an increased DFS in breast cancer patients regardless of receptor status. Consistently, a relationship between tumor-infiltrating plasma cells and improved survival has been reported in non-small cell lung cancer 39.

Mast cells are considered crucial effector cells of the immune system. However, their role in tumorigenesis is controversial, which may be a result of local stromal conditions 40, 41. It is generally believed that mast cells could stimulate growth, neo-angiogenesis and metastasis in tumors, and therefore they are linked to worse outcome. Conversely, some studies have confirmed that mast cells can mobilize T cells and DCs for the regulation of adaptive T-cell mediated immunity 41. In this study, a higher proportion of activated mast cells was associated with an improved OS in HER2-negative tumors. Thus, the function of mast cells in breast cancer patients may be different depending on the tumor subtype. This indicates that mast cells could serve as a potential therapeutic target in HER2-negative tumors.

Finally, the prognostic value of some important immune checkpoint modulators was revealed. The interactions of immune checkpoint modulators can regulate immune responses. Recently, an attractive strategy for immunotherapy has been developed that involves the suppression of immune tolerance by inhibiting the interaction between tumor cells and immune cells 19, 42. Our observations in this study showed that the expression levels of TIM-3, TIGIT, IDO1, ICOS, CTLA4, PD-L1 and PD-L2 were associated with a favorable prognosis in HER2-positive or ER-positive breast cancer. A similar situation has also been reported in breast cancer and other carcinomas, and this may contribute to the insufficient and heterogeneous expression of checkpoint molecules 17 and may be influenced by age, tumor size, histologic grade, nodal metastasis, hormone receptor status, HER2 status, and the extent of TILs 43, 44. Clinical trials have shown that anti-tumor immunotherapies targeting immune checkpoints have achieved considerable success in both positive and negative cancer patients, and this is an argument for the use of immunomodulator inhibiting drugs in breast cancer patients and also indicates that it may be worthwhile to study the relationship between immune checkpoint modulators in TIICs and cancer prognosis.

## Conclusion

In this study, we revealed the complex relationships between infiltrating immune cells and tumor subtypes, clinicopathological parameters and survival in breast cancer. The results showed that a multitude of immune cells were associated with therapeutic responses and prognosis. Moreover, there were large changes in TIIC composition in various subtypes, suggesting the complex involvement of the immune system in breast cancer patients. Based on our analyses, it is worth determining the proportions of M0 macrophages, resting memory CD4^+^ T cells and γδ T cells in all breast cancer patients. Plasma cells, M0 macrophages and regulatory T cells are valuable in ER-positive subtypes, while activated memory CD4^+^ T cells are favorable in terms of DFS in ER- or PR-negative patients or those with a combined subtype. Activated mast cells play a critical role in HER2-negative tumors, but activated NK cells have a significant adverse effect on HER2-positive tumors, as well as patients in the middle and terminal stage of breast cancer. Overall, this study can promote the understanding of immune phenotypes and provide valuable guidance to optimize immunotherapeutic regimens or combined chemoimmunotherapy strategies to achieve improved outcomes for breast cancer patients that are not responsive to existing treatments.

## Figures and Tables

**Table 1:** Primary tumor characteristics

Variable	No. of sample	Valid%
**Age at diagnosis, y**		
≤50	727	43.59%
>50	941	56.41%
**Gender**		
Female	2166	100%
Male	0	0
**Tumor grade**		
1	142	12.24%
2	473	40.78%
3	545	46.98%
**ER status**		
Positive	938	52.64%
Negative	844	47.36%
**HER2 status**		
Positive	251	22.55%
Negative	862	77.45%
**PR status**		
Positive	741	42.83%
Negative	989	57.17%

## Abbreviations

TIICs: tumor-infiltrating immune cells
TILs: tumor-infiltrating lymphocytes
TAMs: tumor-associated macrophages
ER: estrogen receptor
PR: progesterone receptor
HER2: human epidermal growth factor receptor 2
TNBC: triple-negative breast cancer
OS: overall survival
DFS: disease-free survival
HR: hazard ratio
CI: confidence interval
CTLA4: T-lymphocyte-associated protein 4
PD-1: programmed death-1
PD-L1: programmed death-ligand 1
Tfh: follicular helper T cells
NK: natural killer cells
TNM: tumor node metastasis

## References

[1] Siegel RL, Miller KD, Jemal A (2019). Cancer statistics, 2019. CA Cancer J Clin.

[2] Early Breast Cancer Trialists' Collaborative Group (EBCTCG), Davies C, Godwin J, Gray R, Clarke M, Cutter D (2011). Relevance of breast cancer hormone receptors and other factors to the efficacy of adjuvant tamoxifen: patient-level meta-analysis of randomised trials. Lancet.

[3] Berry DA, Cronin KA, Plevritis SK, Fryback DG, Clarke L, Zelen M (2005). Effect of screening and adjuvant therapy on mortality from breast cancer. N Engl J Med.

[4] Bense RD, Sotiriou C, Piccart-Gebhart MJ, Haanen JBAG, van Vugt MATM, de Vries EGE (2017). Relevance of tumor-infiltrating immune cell composition and functionality for disease outcome in breast cancer. J Natl Cancer Inst.

[5] Xu Y, Lan S, Zheng Q (2018). Prognostic significance of infiltrating immune cell subtypes in invasive ductal carcinoma of the breast. Tumori.

[6] Zhou R, Zhang J, Zeng D, Sun H, Rong X, Shi M (2019). Immune cell infiltration as a biomarker for the diagnosis and prognosis of stage I-III colon cancer. Cancer Immunol Immunother.

[7] Denkert C, von Minckwitz G, Darb-Esfahani S, Lederer B, Heppner BI, Weber KE (2018). Tumour-infiltrating lymphocytes and prognosis in different subtypes of breast cancer: a pooled analysis of 3771 patients treated with neoadjuvant therapy. Lancet Oncol.

[8] Desmedt C, Haibe-Kains B, Wirapati P, Buyse M, Larsimont D, Bontempi G (2008). Biological processes associated with breast cancer clinical outcome depend on the molecular subtypes. Clin Cancer Res.

[9] Denkert C, Loibl S, Noske A, Roller M, Müller BM, Komor M (2010). Tumor-associated lymphocytes as an independent predictor of response to neoadjuvant chemotherapy in breast cancer. J Clin Oncol.

[10] Denkert C, von Minckwitz G, Brase JC, Sinn BV, Gade S, Kronenwett R (2015). Tumor-infiltrating lymphocytes and response to neoadjuvant chemotherapy with or without carboplatin in human epidermal growth factor receptor 2-positive and triple-negative primary breast cancers. J Clin Oncol.

[11] Loi S, Michiels S, Salgado R, Sirtaine N, Jose V, Fumagalli D (2014). Tumor infiltrating lymphocytes are prognostic in triple negative breast cancer and predictive for trastuzumab benefit in early breast cancer: results from the FinHER trial. Ann Oncol.

[12] Loi S, Sirtaine N, Piette F, Salgado R, Viale G, Van Eenoo F (2013). Prognostic and predictive value of tumor-infiltrating lymphocytes in a phase III randomized adjuvant breast cancer trial in node-positive breast cancer comparing the addition of docetaxel to doxorubicin with doxorubicin-based chemotherapy: BIG 02-98. J Clin Oncol.

[13] Adams S, Gray RJ, Demaria S, Goldstein L, Perez EA, Shulman LN (2014). Prognostic value of tumor-infiltrating lymphocytes in triple-negative breast cancers from two phase III randomized adjuvant breast cancer trials: ECOG 2197 and ECOG 1199. J Clin Oncol.

[14] Kreike B, van Kouwenhove M, Horlings H, Weigelt B, Peterse H, Bartelink H (2007). Gene expression profiling and histopathological characterization of triple-negative/basal-like breast carcinomas. Breast Cancer Res.

[15] Choi J, Gyamfi J, Jang H, Koo JS (2018). The role of tumor-associated macrophage in breast cancer biology. Histol Histopathol.

[16] AiErken N, Shi HJ, Zhou Y, Shao N, Zhang J, Shi Y (2017). High PD-L1 expression is closely associated with tumor-infiltrating lymphocytes and leads to good clinical outcomes in Chinese triple negative breast cancer patients. Int J Biol Sci.

[17] Alsaab HO, Sau S, Alzhrani R, Tatiparti K, Bhise K, Kashaw SK (2017). PD-1 and PD-L1 checkpoint signaling inhibition for cancer immunotherapy: mechanism, combinations, and clinical outcome. Front Pharmacol.

[18] Zamarron BF, Chen W (2011). Dual roles of immune cells and their factors in cancer development and progression. Int J Biol Sci.

[19] Sun YS, Zhao Z, Yang ZN, Xu F, Lu HJ, Zhu ZY (2017). Risk factors and preventions of breast cancer. Int J Biol Sci.

[20] Liu X, Wu S, Yang Y, Zhao M, Zhu G, Hou Z (2017). The prognostic landscape of tumor-infiltrating immune cell and immunomodulators in lung cancer. Biomed Pharmacother.

[21] Newman AM, Liu CL, Green MR, Gentles AJ, Feng W, Xu Y (2015). Robust enumeration of cell subsets from tissue expression profiles. Nat Methods.

[22] Ali HR, Chlon L, Pharoah PD, Markowetz F, Caldas C (2016). Patterns of immune infiltration in breast cancer and their clinical implications: A gene-expression-based retrospective study. PLoS Med.

[23] Dai X, Xiang L, Li T, Bai Z (2016). Cancer hallmarks, biomarkers and breast cancer molecular subtypes. J Cancer.

[24] Dai X, Li T, Bai Z, Yang Y, Liu X, Zhan J (2015). Breast cancer intrinsic subtype classification, clinical use and future trends. Am J Cancer Res.

[25] Zitvogel L, Tanchot C, Granier C, Tartour E (2013). Following up tumor-specific regulatory T cells in cancer patients. Oncoimmunology.

[26] Tanaka A, Sakaguchi S (2017). Regulatory T cells in cancer immunotherapy. Cell Res.

[27] Curiel TJ, Coukos G, Zou L, Alvarez X, Cheng P, Mottram P (2004). Specific recruitment of regulatory T cells in ovarian carcinoma fosters immune privilege and predicts reduced survival. Nat Med.

[28] Su S, Liao J, Liu J, Huang D, He C, Chen F (2017). Blocking the recruitment of naive CD4^+^ T cells reverses immunosuppression in breast cancer. Cell Res.

[29] Chanmee T, Ontong P, Konno K, Itano N (2014). Tumor-associated macrophages as major players in the tumor microenvironment. Cancers (Basel).

[30] Chao J, Zhang Y, Du L, Zhou R, Wu X, Shen K (2017). Molecular mechanisms underlying the involvement of the sigma-1 receptor in methamphetamine-mediated microglial polarization. Sci Rep.

[31] Zhang R, Li F, Li H, Yu J, Ren X (2014). The clinical significance of memory T cells and its subsets in gastric cancer. Clin Transl Oncol.

[32] Lalvani A, Brookes R, Hambleton S, Britton WJ, Hill AV, McMichael AJ (1997). Rapid effector function in CD8^+^ memory T cells. J Exp Med.

[33] Novy P, Quigley M, Huang X, Yang Y (2007). CD4 T cells are required for CD8 T cell survival during both primary and memory recall responses. J Immunol.

[34] Hwang ML, Lukens JR, Bullock TN (2007). Cognate memory CD4^+^ T cells generated with dendritic cell priming influence the expansion, trafficking, and differentiation of secondary CD8^+^ T cells and enhance tumor control. J Immunol.

[35] Crome SQ, Lang PA, Lang KS, Ohashi PS (2013). Natural killer cells regulate diverse T cell responses. Trends Immunol.

[36] Donnelly RP, Loftus RM, Keating SE, Liou KT, Biron CA, Gardiner CM (2014). mTORC1-dependent metabolic reprogramming is a prerequisite for NK cell effector function. J Immunol.

[37] Poznanski SM, Barra NG, Ashkar AA, Schertzer JD (2018). Immunometabolism of T cells and NK cells: metabolic control of effector and regulatory function. Inflamm Res.

[38] Nera KP, Kylaniemi MK, Lassila O (2015). Regulation of B cell to plasma cell transition within the follicular B cell response. Scand J Immunol.

[39] Lohr M, Edlund K, Botling J, Hammad S, Hellwig B, Othman A (2013). The prognostic relevance of tumour-infiltrating plasma cells and immunoglobulin kappa C indicates an important role of the humoral immune response in non-small cell lung cancer. Cancer Lett.

[40] Onnes MC, Tanno LK, Elberink JN (2016). Mast cell clonal disorders: classification, diagnosis and management. Curr Treat Options Allergy.

[41] Ammendola M, Sacco R, Sammarco G, Luposella M, Patruno R, Gadaleta CD (2016). Mast cell-targeted strategies in cancer therapy. Transfus Med Hemother.

[42] Huang A, Cao S, Tang L (2017). The tumor microenvironment and inflammatory breast cancer. J Cancer.

[43] Schalper KA, Velcheti V, Carvajal D, Wimberly H, Brown J, Pusztai L (2014). In situ tumor PD-L1 mRNA expression is associated with increased TILs and better outcome in breast carcinomas. Clin Cancer Res.

[44] Kong P, Wang J, Song Z, Liu S, He W, Jiang C (2019). Circulating lymphocytes, PD-L1 expression on tumor-infiltrating lymphocytes, and survival of colorectal cancer patients with different mismatch repair gene status. J Cancer.

